# All-in-One CO_2_ Capture and Transformation: Lessons from Formylmethanofuran Dehydrogenases

**DOI:** 10.1021/acs.accounts.4c00623

**Published:** 2024-11-25

**Authors:** Olivier N. Lemaire, Tristan Wagner

**Affiliations:** Max Planck Institute for Marine Microbiology, Celsiusstraße 1, 28359 Bremen, Germany

## Abstract

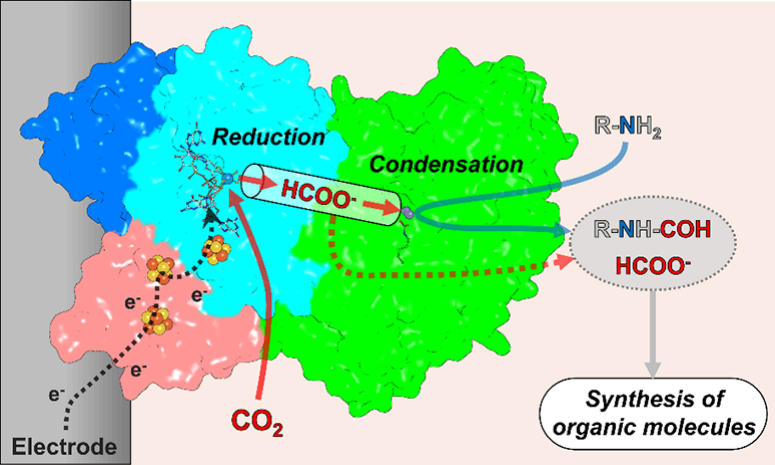

Carbon-one-unit (C1) feedstocks are generally used in the chemical synthesis of organic molecules, such as solvents, drugs, polymers, and fuels. Contrary to the dangerous and polluting carbon monoxide mostly coming from fossil fuels, formate and formamide are attractive alternative feedstocks for chemical synthesis. As these are currently mainly obtained from the oil industry, novel synthetic routes have been developed based on the transformation of the greenhouse gas CO_2_. Such developments are motivated by the urgent need for carbon chemical recycling, leading to a sustainable future. The inert nature of CO_2_ represents a challenge for chemists to activate and specifically convert the molecule through an affordable and efficient process. The chemical transformation could be inspired by biological CO_2_ activation, in which highly specialized enzymes perform atmospheric CO_2_ fixation through relatively abundant metal catalysts. In this Account, we describe and discuss the potential of one of the most efficient biological CO_2_-converting systems: the formylmethanofuran dehydrogenase (abbreviated as FMD).

FMDs are multienzymatic complexes found in archaea that capture CO_2_ as a formyl group branched on the amine moiety of the methanofuran (MFR) cofactor. This overall reaction leading to formyl−MFR production does not require ATP hydrolysis as compared to the CO_2_-fixing microbes relying on the reductive Wood–Ljungdahl pathway, highlighting a different operative mode that saves cellular energy. FMD reaction represents the entry point in hydrogenotrophic methanogenesis (H_2_ and CO_2_ dependent or formate dependent) and operates in reverse in other methanogenic pathways and microbial metabolisms. Therefore, FMD is a key enzyme in the planetary carbon cycle. After decades of investigations, recent studies have provided a description of the FMD structure, reaction mechanism, and potential for the electroreduction of CO_2_, to which our laboratory has been actively contributing.

FMD is an “all-in-one” enzyme catalyzing a redox-active transformation coupled to a redox-neutral transformation at two very different metal cofactors where new C–H and C–N bonds are made. First, the principle of the overall reaction consisting of an exergonic CO_2_ reduction coupled with an endergonic formate condensation on MFR is resumed. Then, this Account exposes the molecular details of the active sites and provides an overview of each catalytic mechanism. It also describes the natural versatility of electron-delivery modules fueling CO_2_ reduction and extends it to the possibilities of using artificial systems such as electrodes.

A perspective concludes on how the mechanistic of FMD could be applied to produce CO_2_-based chemical intermediates to synthesize organic molecules. Indeed, through its biochemical properties, the enzyme opens opportunities for CO_2_ electroreduction to generate molecules such as formate and formamide derivatives, which are all intermediates for synthesizing organic compounds. Transferring the chemical knowledge acquired from these biological systems would provide coherent models that can lead to further development in the field of synthetic biology and bio-inspired synthetic chemistry to perform large-scale CO_2_ conversion into building blocks for chemical synthesis.

## Key References

- SahinS.; LemaireO. N.; BelhamriM.; KurthJ. M.; WelteC. U.; WagnerT.; MiltonR. D.Bioelectrocatalytic CO_2_ Reduction by Mo-Dependent Formylmethanofuran Dehydrogenase. Angew. Chem., Int. Ed.2023, 62 ( (45), ), e20231198110.1002/anie.20231198137712590.^1^ This is the first characterization of FMD as biocatalysts for CO_2_ electroreduction by direct coupling to an electrode. This work demonstrated that the specific architecture of these enzymes favors formate accumulation, making them more adapted for applied applications.
- LemaireO. N.; WegenerG.; WagnerT.Ethane-oxidizing archaea couple CO_2_ generation to F_420_ reduction. Nat. Commun.2024, 15 ( (1), ), 906539433727 10.1038/s41467-024-53338-7PMC11493965.^2^ This work presents the third structure of an FMD, describing a novel organization and enzymatic coupling. It is the first picture of an FMD generating CO_2_ in a physiological context.

## 1 Introduction

One-carbon unit (C1) molecules are building blocks for synthesizing organic chemicals such as solvents, drugs, textiles, polymers, detergents, food additives, or biofuels. These C1 feedstocks include carbon monoxide (CO), methanol, formate, or formamide,^3−6^ commonly used to generate reactive groups in organic molecules, allowing further reactions.^4,7^ CO is one of the most important industrial C1 sources because of its utilization in the Fischer–Tropsch process and its conversion to other C1 molecules or carbonylated transition metals involved in a wide range of chemical syntheses.^4,8^ Mainly obtained via the gasification of fossil fuels, CO has major drawbacks that hamper its storage, transport, and handling due to its reactivity, toxicity, and explosiveness. Extensive efforts are being made to use alternative C1 sources, enhanced by the current context of the rarefaction of fossil fuels and the climate crisis. Recent studies demonstrated that formate (referring here to both formate and formic acid) and formamide (e.g., through CO or formate generation) can replace CO in the chemical synthesis of organic molecules^3−5^ or be used as a storage material for energy or carbon sources.^4,9,10^ Formate or formamide production from CO_2_ is being intensely investigated to develop new strategies for promoting a carbon cycling economy.

**Figure 1:**
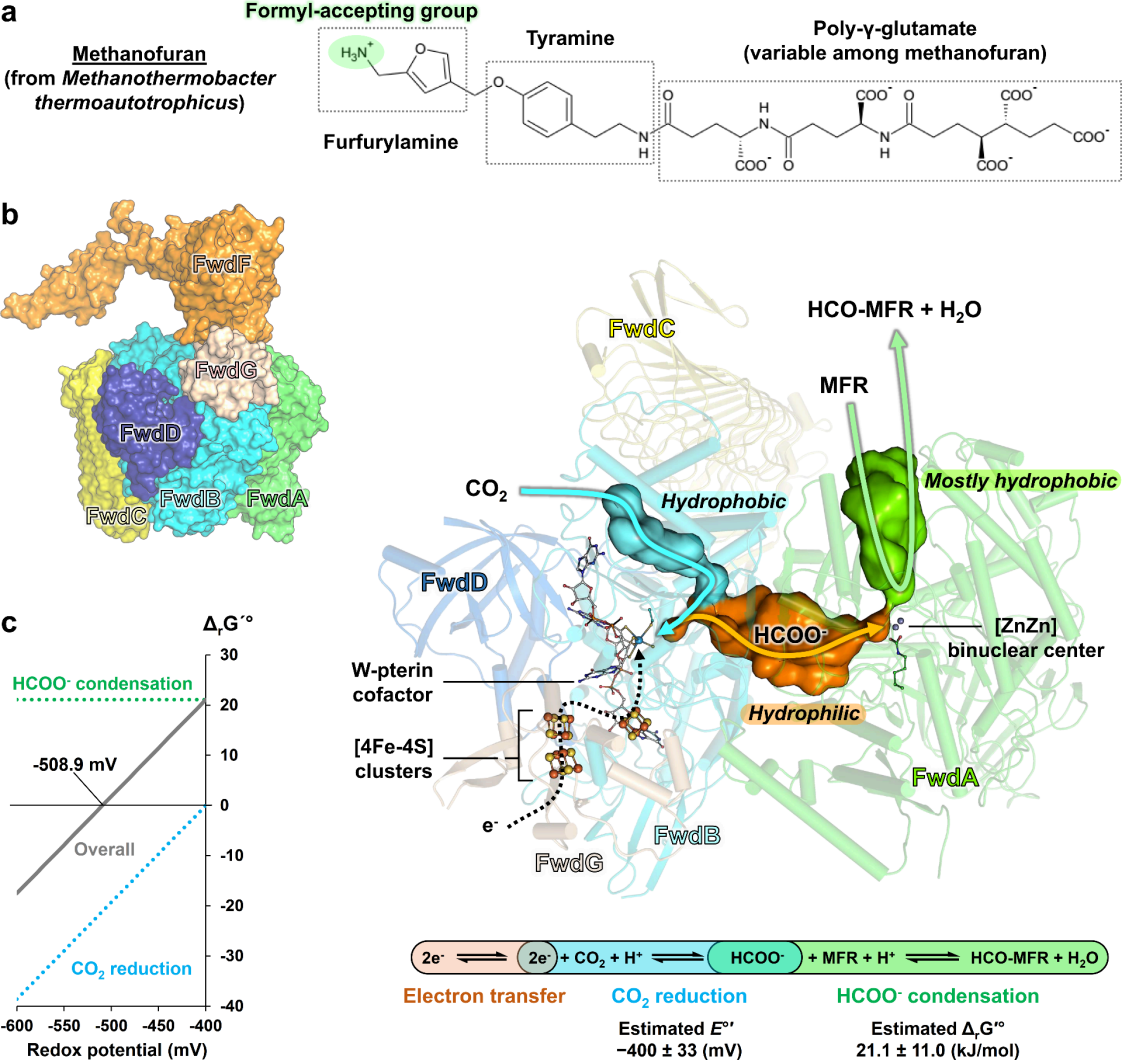
Substrate, internal trafficking, and general reaction mechanism in FMDs. (a) MFR structure. (b) FMD overall architecture. The subunits of the *M. wolfei* structure (PDB 5T5M) are shown as surfaces in the inset. The central figure depicts the enzyme as transparent cartoons with (metallo)cofactors in balls and sticks with FwdF omitted for clarity. Carbon, oxygen, nitrogen, sulfur, phosphorus, iron, and tungsten are colored white/green, red, blue, yellow, light orange, orange, and gray-blue, respectively. The internal cavities displayed as surfaces were predicted by the HOLLOW program,^22^ ignoring the Lys64 side chain (see Figure 3). Blue, orange, and green lines schematize the CO_2_, formate, and MFR internal transfers, respectively. The enzymatic reactions shown with *E°′* and Δ_r_G′° come from eQuilibrator.^23^ (c) The difference in Gibbs free energy of both half-reactions and their addition (“Overall”) depends on the redox potential (at pH 6.9, assumed to be physiological^24^).

CO_2_ is the most oxidized form of carbon and is an abundant C1 molecule. It is a linear molecule stable under atmospheric conditions.^11^ To destabilize CO_2_ and drive its reduction to CO or formate, chemical processes necessitate intensive energy inputs (e.g., low electron redox potentials and high temperature and pressure). On the other hand, CO_2_ is a common substrate for living organisms that reduce it under normal temperature and pressure conditions in aqueous solutions.^11,12^ The microbial reductive acetyl–Coenzyme A pathway is the most energetically efficient CO_2_-fixing biological process reported so far.^12−14^ In this pathway, CO_2_ is reduced to formate and then condensed as a formyl group on a C1 carrier.^14^ In bacteria, distinct enzymes catalyze both reactions independently and couple the formate condensation to ATP hydrolysis.^14^ In contrast, archaea generating biological methane (called methanogens) rely on formylmethanofuran dehydrogenases (FMDs) to directly capture CO_2_ as a bound formyl group on the methanofuran (MFR; Figure 1a) in an ATP-independent reaction.^15^ FMDs are metalloenzymes composed of a formate dehydrogenase bound to an amidohydrolase. The formate dehydrogenase is flanked by an electron-transferring system that can form relatively large oligomers. The overall reaction of FMD depends on several metallic cofactors (i.e., Mo/W–pterin, [Zn–Zn] dinuclear site, and [4Fe–4S] clusters), with some being oxygen-sensitive. FMDs are central to the global carbon cycle, because they are crucial enzymes in a broad diversity of methanogens and archaeal alkane oxidizers. Exciting breakthroughs in recent years accelerated our understanding of this all-in-one enzyme and its electrochemical properties, revealing an attractive catalyst to convert CO_2_ into different organic molecules.^1,2,15−17^

This Account reviews and discusses the recent data gathered on FMD, detailing its composition, organization, and reaction mechanism, while presenting its advantages for CO_2_ conversion to C1 feedstocks. We also propose tools and strategies for using them as a biocatalyst or as a template for developing bio-inspired synthetic catalysts.

## 2 FMDs as CO_2_ Converters

### 2.1 Two-Step Reaction in a Single Protein Complex

The CO_2_ conversion to formyl–MFR during methanogenesis was demonstrated in 1985,^18^ the responsible enzyme being purified 4 years later.^19^ The reaction mechanism of the enzyme has, however, been puzzling for decades. Several studies could not detect a direct formate production from CO_2_, leading to a mechanism depending on carboxy–MFR as a reaction intermediate.^18,20,21^ The proposed scenario initiates with the nucleophilic attack on CO_2_, occurring spontaneously or enzymatically by FMD to generate a carboxy–MFR. This latter would be enzymatically reduced to formyl–MFR. The supposed carboxy–MFR intermediate has never been detected, which was supposed to be due to its instability.^18^ Three decades after its identification, the overall picture of the reaction became clearer when T.W. (here as corresponding author) solved the crystal structure of the tungsten-dependent FMD (Fwd complex) from the hydrogenotrophic methanogen *Methanothermobacter wolfei* (*M. wolfei*) in the group of Seigo Shima.^16^

The native enzyme was purified and crystallized in the absence of oxygen.^15,16^ The structure depicts the enzyme assembly (Figure 1b). The core is composed of the CO_2_-reducing module (FwdBD) bound to an amidohydrolase module (FwdA) and flanked by an electron-donating module (FwdFG). The interface bridging both catalytic components is strengthened by a β-helicoidal subunit (FwdC), which stabilizes an internal cavity connecting the two active sites (Figure 1b). This interaction is reminiscent of glutamate synthases in which a similar β-helicoidal domain binds on the amidotransferase and synthase domains to stabilize an internal ammonia channel.^25^ The analogy with the glutamate synthase pushed the authors to propose a tunneling-dependent mechanism decoupled in the following steps: (i) The CO_2_ is guided by a hydrophobic channel to the pterin cofactor in the BD subunits catalyzing its reduction to formate; (ii) the polar formate is trapped in a hydrophilic internal cavity connecting the CO_2_-reducing site to a binuclear [Zn–Zn] center harbored by the amidohydrolase subunit; (iii) the [Zn–Zn] center catalyzes the condensation of formate and the amino group of MFR to generate a water molecule and formyl–MFR.^15,16^ The amidohydrolase module hence operates in the reverse direction compared to homologues canonically involved in hydrolysis reactions. A two-electron transfer through the electron-donating module reduces pterin, preparing the next catalytic cycle. The authors assumed that formate and not formic acid is the intermediate species due to the analogy of reaction with formate dehydrogenases (see Section 2.2), the internal polar cavity filled with water and without acidic residues, and the positively charged lysine at the “end” of the cavity that would interact with the negatively charged formate (see Section 2.3).

Following the proposed mechanism, the endergonic reaction of formate condensation on the MFR would be achieved by accumulating a large excess of formate in the internal cavity (*K*′_eq_ = 2.0 × 10^–4^, pH 6.9). This implies that the overall reaction combining formate generation and its condensation will be unfavorable at the midpoint standard redox potential for the CO_2_/formate couple (i.e., −0.4 V). However, a redox potential below −0.51 V (see Section 3.1) allows sufficient accumulation of the formate to overcome the second reaction catalyzed by the amidohydrolase, as illustrated in Figure 1c. Here, the change in Gibbs free energy would be zero, and the local excess of formate concentration is sufficient to promote formyl–MFR synthesis. Accordingly, the redox midpoint potential of the reaction was experimentally estimated at −0.53 V,^15,26^ significantly lower than the potential of the formate/CO_2_ couple. The small volume of the internal cavity would stimulate the rapid increase of the local formate accumulation, and the fast consumption of formyl−MFR through the methanogenesis pathway will also favor the overall reaction.

Three other homologous systems were structurally characterized later. The distant bacterial formyltransferase/hydrolase complex studied by the corresponding author,^27^ the supercomplex from the methanogen *Methanospirillum hungatei* (*Mp. hungatei*) unveiled by the Shima and Murphy’s groups,^17^ and the FMD from an ethane-oxidizing microbial consortium from our group.^2^ Together, these investigations describe the functional diversity of this enzyme used for a wide range of microbial metabolism.

### 2.2 CO_2_ Reduction at the W/Mo–Pterin Module

The CO_2_-reducing module (formed by the B and D subunits) is structurally similar to the metal-dependent formate dehydrogenase (FDH, formed by a unique polypeptide). The catalyst of the reaction, a metallopterin cofactor, is carried at the interface of the B and D subunits. The absence of the D subunit and remodeling of the loops on the B subunit provokes the loss of the metallopterin in the homologue from *Methylorubrum extorquens* (*M. extorquens*), abolishing its ability to oxidize formate.^27^

**Figure 2:**
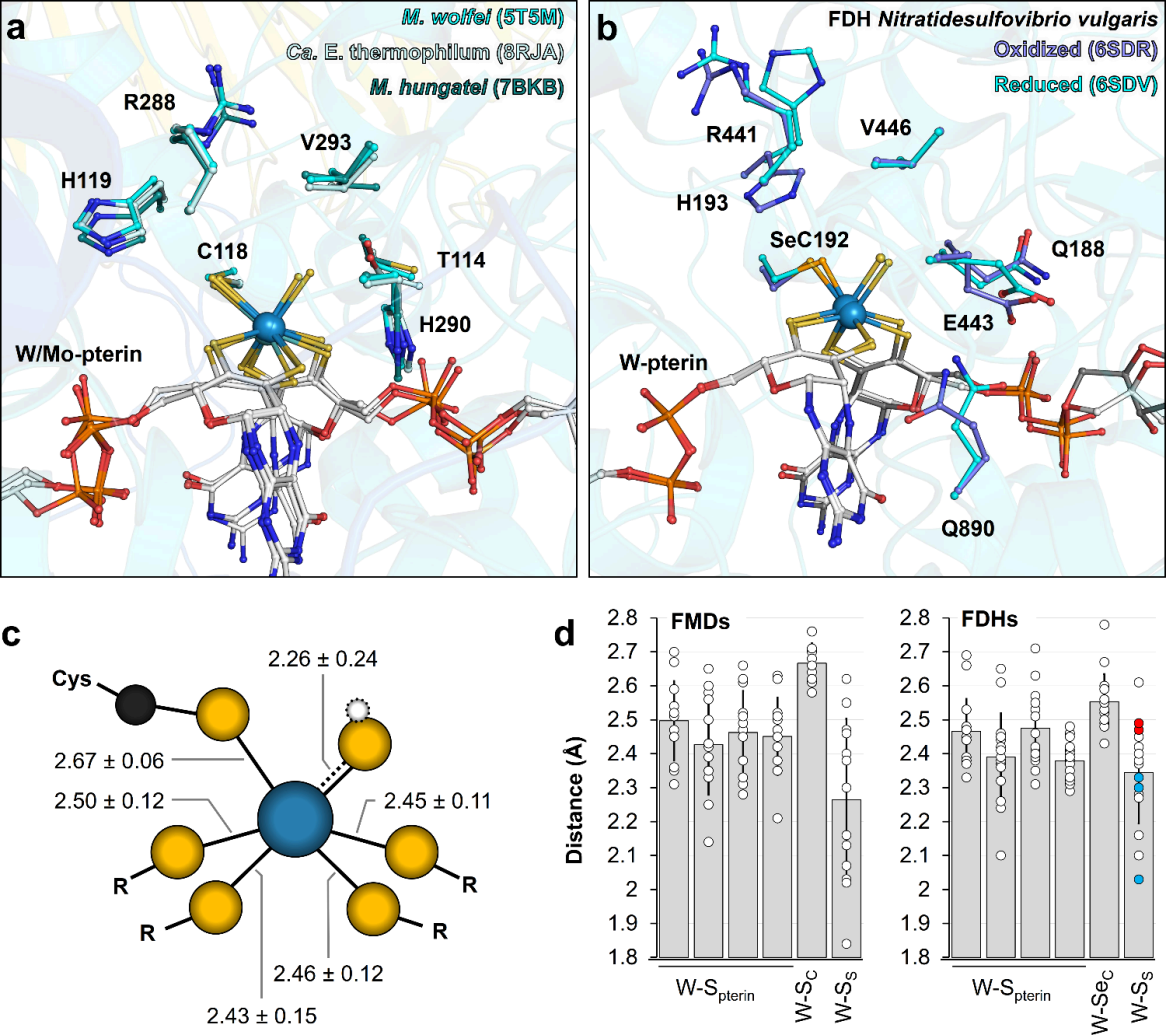
CO_2_ reduction center. FMDs (a) and FDHs (b) active sites. Structures are in transparent cartoons with pterin cofactor and surrounding residues (*M. wolfei* numbering) shown in balls and sticks and colored as in Figure 1 with Mo (only for PDB 7BKB) colored light blue. The axial ligand was not modeled in the cryo-EM structures of *M. hungatei* (PDB 7BKB), probably due to the relatively low resolution.^17^ (c) Tungsten coordination in crystallographic structures of FMDs (PDBs 5T5M/5T5I/5T61/8RJA). A white dotted sphere indicates the putative hydrogen atom of the sulfhydryl ligand. Distances (in Å) indicate the average and standard deviations calculated from all FMD-reported crystallographic structures, as shown in panel d. (d) Average and individual measurements of the bond distances of Mo/W in the FMDs (left) and FDHs (right) crystal structures. The distances between the Mo/W and the S from both dithiolene (W–S_pterin_), the S/Se of the proteinogenic (seleno)cysteine ligand (W–S_c_/W–Se_c_), and the sulfide/sulfhydryl ligands (W–S_S_) are labeled. PDB codes: 5T5M/5T5I/5T61/8RJA for FMDs and 1KQF/1KQG/1HOH/6SDR/6SDV/7Z5O/8BQG/8BQH/8BQI/8BQJ/8BQK/8BQL/8CM4/8CM5/8CM6/8RCG for FDHs analyzed structures. The significant variations in the distance between the different models might result from a mixture of reduced and oxidized states^30^ or from radiation damage, as highlighted by the differences in the FDH reduced (red dots) versus oxidized (blue dots) state.

As for most FDHs, FMDs are sensitive to oxygen because of their W- (Fwd complexes) or Mo- (Fmd complexes) containing pterin (here, referred to as W/Mo–pterin).^15^ Differentiating the metal composition based on protein sequence is not feasible and requires experimental evidence.^15^ Organisms can encode one or several isoforms specific for Mo, W, or even Se (the latter being an axial ligand of the metallopterin; see below) probably to cope with environmental metal bioavailability. Mo or W are bound to the pterin and enzyme via trigonal-prismatic coordination comprising six sulfur atoms: four from the dithiolene groups of the bis-pyranopterin guanosine dinucleotide (bis-PGD), one from a proteinogenic cysteine residue from the B subunit, and a last atom from a sulfido/sulfhydryl axial ligand (Figure 2). The sulfur from the cysteine can be exchanged by the selenium of selenocysteine, recognizable from the gene sequence by an internal stop codon.^15^ Bis-PGD is usually perfectly conserved except for one case found in the *Methanosarcinales* order, where variations of nucleotides have been found,^28^ which is not thought to impact catalysis.

The axial sulfido/sulfhydryl ligand is proposed to be a hallmark of this family as it was shown to be catalytically relevant in FDHs (Figures 2a–c).^29^ The W–S bond length observed in the crystallographic structures suggests a double-bonded sulfido rather than a sulfhydryl group in some models, as in FDHs. The distance between the catalytic metal and proteinogenic sulfur ligands is statistically longer than other W–S bonds in FMDs, which may suggest an electronic displacement. Such an observation is less clear in FDH structures, putatively, because all reported structures present a selenocysteine.

The residues surrounding the catalytic metal are conserved between FMDs and FDHs with some variability (e.g., His290 in *M. wolfei* substituted by glutamate in FDH, Figures 2a,b). Based on the conservation of the metallocofactor and its environment, it is assumed that FMDs and FDHs share the same catalytic principle.

Despite being extensively studied for years, the reaction mechanism of FDHs is still debated.^11,30−38^ One proposed scenario suggests the replacement of either the cysteine or sulfido ligands by the CO_2_ molecule as metal–ligand, with the putative formation of a thiocysteine intermediate.^11,35,37^ Alternatively, the six S (or 5S/Se) ligands could be conserved during catalysis, and the CO_2_ reduction would occur in the second coordination sphere of the catalytic metal.^30−33,38,39^ The conserved residues near the metal would act as proton donors for the Cys/SeCys and the CO_2_ or trigger the “activation” of CO_2_,^11^ depending on the selected reaction model. CO_2_ activation consists of bending the molecule structure, increasing the length of the C–O bond,^11^ and facilitating nucleophilic attack. The equivalent position of His119 was proposed to act as a gating mechanism in FDHs.^32,39^ The side chain switches position depending on the redox state of the enzyme (His193 in Figure 2b) to control the access of CO_2_ or formate to the active site. For instance, when the enzyme is reduced, the formate channel is sterically hindered by the imidazole side chain.^39^ If the gating mechanism is conserved in FMDs, then the side chain position in the crystallographic structures would indicate an oxidized cofactor (Figure 2a).

Protons must be accessible from the solvent to allow for fast and efficient protonation. In his initial study, the corresponding author proposed a proton channel in the enzyme from *M. wolfei*, reminiscent of the one from FDH.^16^

### 2.3 Formate Condensation at a Metallic Binuclear Site

**Figure 3:**
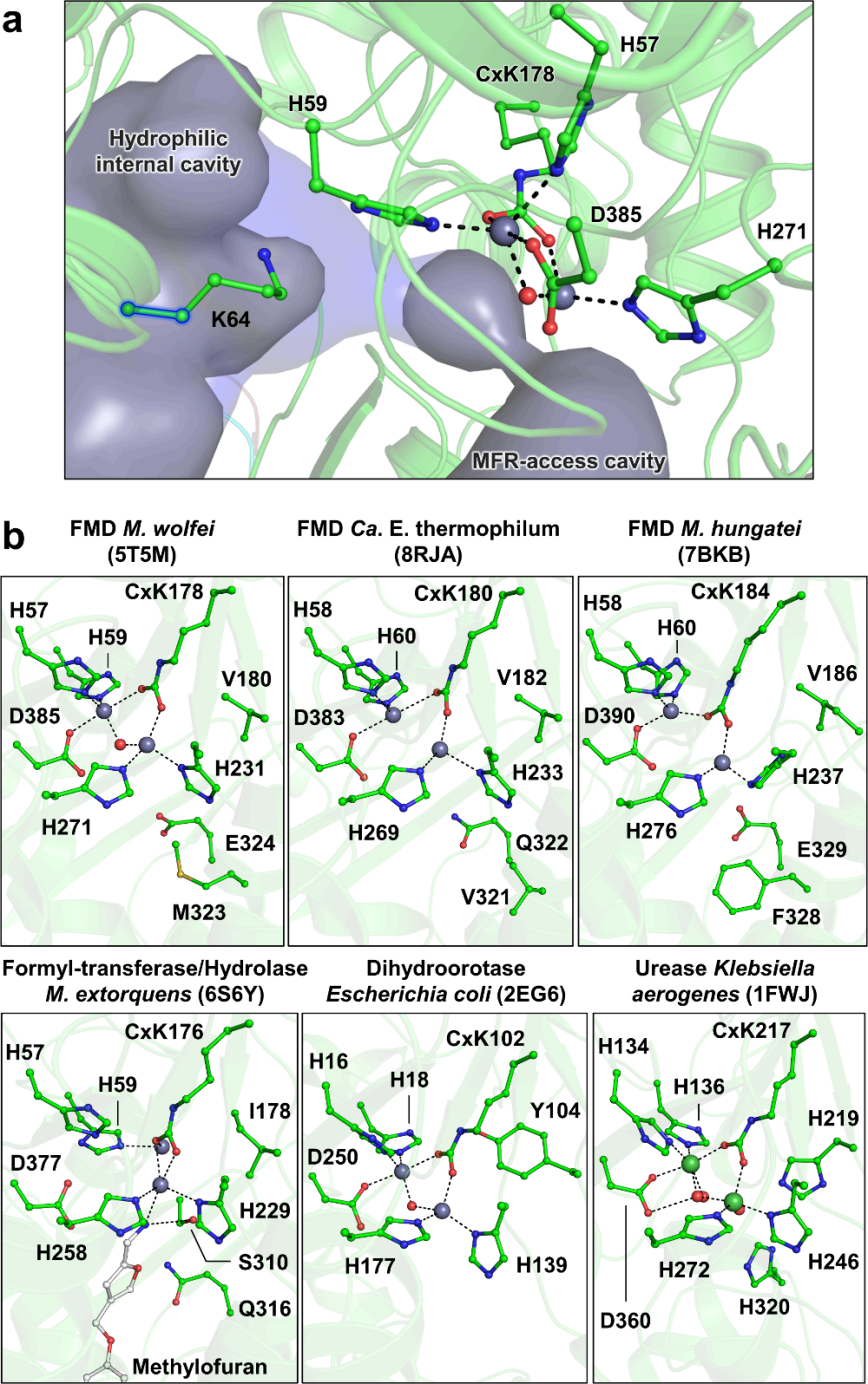
Organization of the amidohydrolase active site. (a) Structure of the FMD of *M. wolfei* (PDB 5T5M) used for cavity determination by HOLLOW with the entire (gray surface) or truncated (transparent blue surface, highlighted with a blue glow) Lys64 side chain. (b) Active site of amidohydrolase in FMDs and related complexes. The structures are shown as transparent cartoons with the catalytic Zn/Ni, bound water, and surrounding residues shown as balls and sticks and colored as in Figure 1. Nickel atoms are colored dark green, and carbons of the methylofuran (an MFR analog from *M. extorquens*(27)) are colored white. Dashed lines represent stabilizing interactions.

The formate produced is released in the hydrophilic cavity connected to the catalytic chamber of subunit A. The access to the amidohydrolase active site seems to be partially hindered by a conserved lysine (Lys64 in *M. wolfei*, harbored on subunit A) in the described structures (Figure 3a). As the orientation of the lysine side chain would sequester formate in the internal cavity, one could assume a “gatekeeper” role of this residue. While speculative, such a gating system would be advantageous in preventing the escape of formate in the absence of MFR, allowing its accumulation in the cavity.^1^

**Figure 4:**
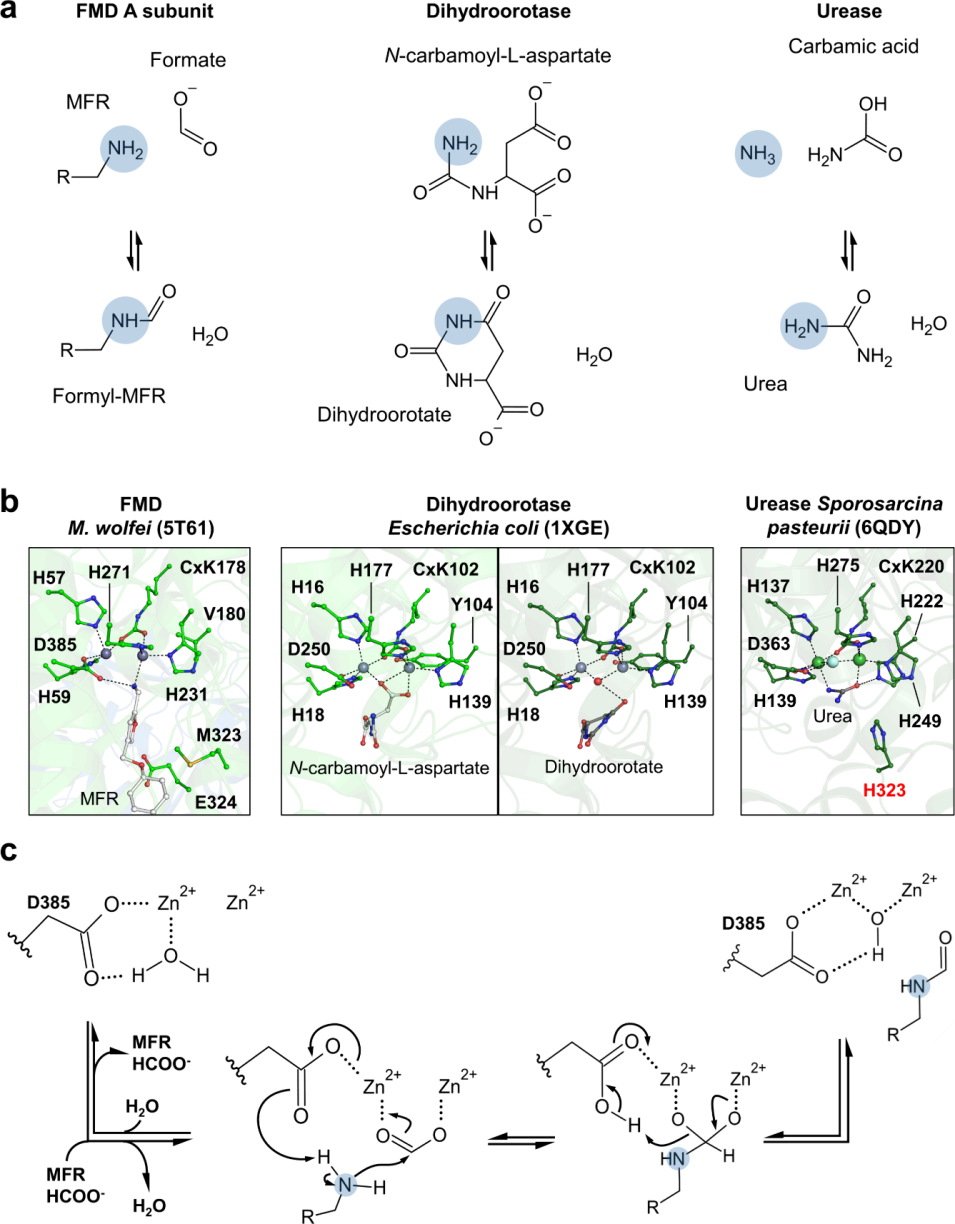
Substrate/product bound crystal structures of amidohydrolases and proposed reaction mechanism of FMD A subunit. (a) Schematic representation of the catalyzed reaction. The MFR molecule (Figure 1a) was simplified as R. (b) Structures displayed in the same fashion as in Figure 3b. Catalytic Zn/Ni, substrates, bound water, fluorine atom (in a light cyan ball), and surrounding residues are shown as balls and sticks. Dashed lines represent stabilizing interactions. The His323, which is supposed to stabilize the ammonium ion after hydrolysis in urease,^40^ is labeled in red. (c) Proposed reaction mechanism of FMD based on dihydoorotase.^41^ Panels a and c: The reactive amino groups are highlighted by a filled blue circle. Molecules were drawn by using ChemSketch.

The A subunit belongs to the amidohydrolases superfamily (Figure 3b) that catalyzes metal-dependent amide hydrolysis or the reverse condensation reaction of different substrates (Figure 4a). However, the A subunit has a low sequence and structural similarity with homologues due to the acquisition of structural traits to assemble with the BC subunits and scaffold the MFR binding platform. The family gathers enzymes harboring [Zn–Zn] or [Ni–Ni] centers. The binuclear sites are systematically coordinated by four histidines, an aspartate, and a carboxylysine (a post-translationally modified lysine), perfectly conserved in the studied FMDs (Figure 3b). Based on the active site conservation, the FMD reaction mechanism has been proposed to be homologous to amidohydrolases. This assumption has been reinforced by the crystal structure of FMD from *M. wolfei* with a bound MFR positioned in the vicinity of a conserved aspartate as observed for substrates in homologues (Figure 4b). The reactive amino group of MFR has a basicity similar to ammonia recognized by ureases (estimated from the furfurylamine p*K*_a_).^40^ However, because of its difference in metals (i.e., Zn versus Ni) and a key histidine stabilizing the second amino group of the urea substituted by valine or isoleucine in FMD (Figure 4b), the mechanism has rather been derived from the dihydroorotase.^41^ The proposed reaction of formate condensation assisted by the conserved aspartate would proceed in four steps (Figure 4c): (i) Formate stabilization on the [Zn–Zn] binuclear site; (ii) abstraction of a proton of the amino group of MFR by the conserved aspartate; (iii) nucleophilic attack of the deprotonated amine on the formate carbon; (iv) generation of formyl–MFR and a [Zn–Zn] bridging hydroxide anion, later released as water.

## 3 Enzymatic Electrocatalysis of CO_2_ into Added Value Chemicals

### 3.1 Diversity of Electron-Transferring Modules

To pull forward the endergonic formate condensation reaction (Figure 1c), an excess of formate must be accumulated in the internal cavity, which is achieved through the strong reducing power fueling the CO_2_ reduction. Hence, the driving force allowing a thermodynamically favorable overall reaction is the redox potential of the electron donor^15,16^ (see Section 2.1). This also means that electron donors used by FMD systems are more restrictive than those for FDHs.^14^ For instance, fueling CO_2_ reduction via H_2_ oxidation (*E*°′ = −0.414 V^14^) is incompatible with FMD systems, which would require lower potential electron donors such as ferredoxin (physiological redox potential estimated at −0.5 V^14^).

**Figure 5:**
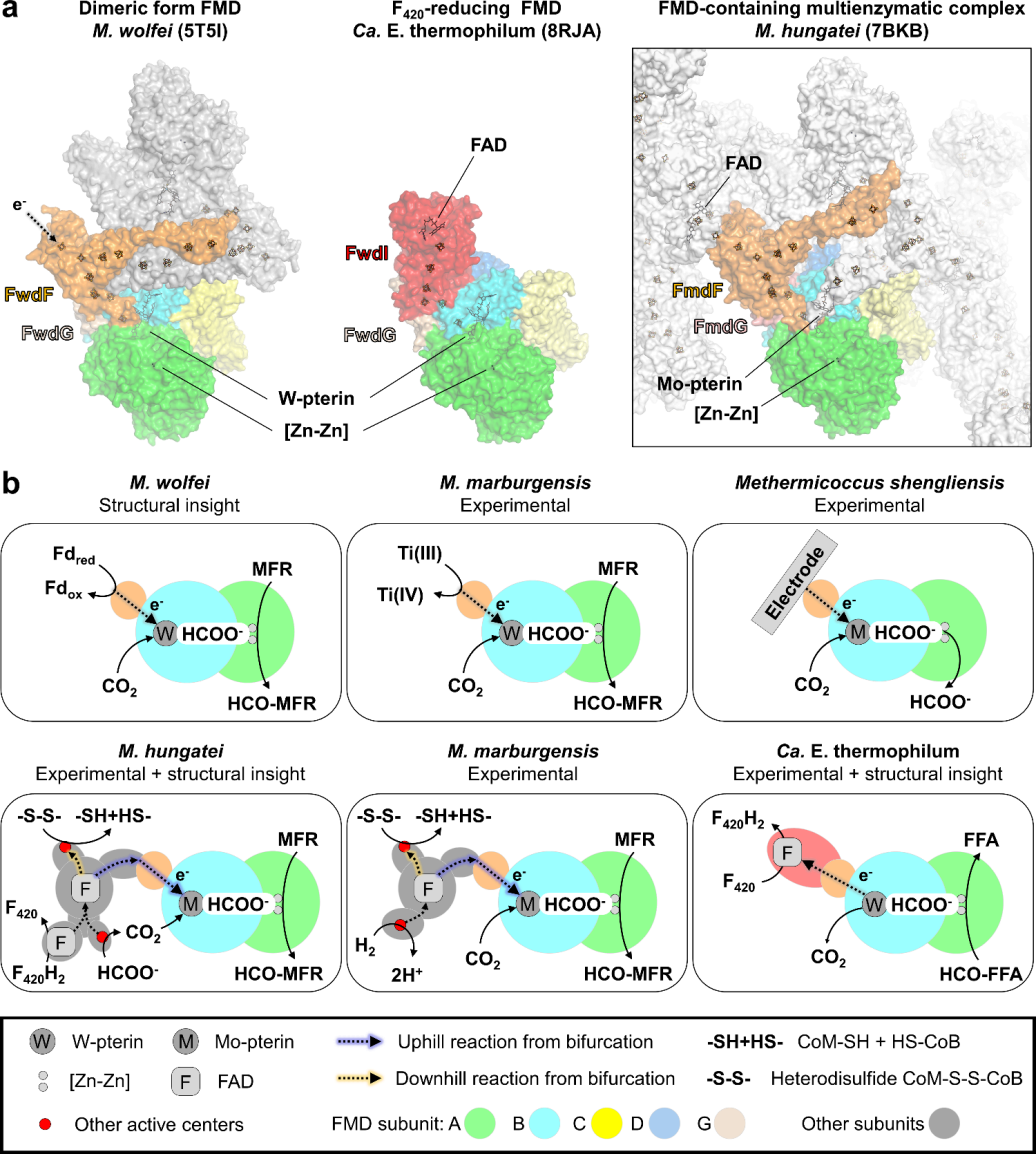
Organization and reactions catalyzed by FMDs. (a) Organization of the structurally characterized FMDs. The dimeric form of *M. wolfei* was chosen over the proposed physiological tetrameric form (PDB 5T61) for graphical reasons. The structures of the complexes are shown as surfaces. Only one set of the subunits is colored as in Figure 1, the rest being gray. Cofactors are shown as balls and sticks. (b) Schematic representation of the different reactions proposed or demonstrated to be catalyzed by the FMDs.^1,2,16,17,42,46^ The amidohydrolase, CO_2_-reducing, and electron-transferring modules are schematized by circles colored green, cyan, and light orange, respectively. Reactions are indicated without considering the stoichiometry. F_420_, FFA, and HCO-FFA stand for cofactor F_420_, *N*-furfurylamine, and *N*-furfurylformamide, respectively.

The first FMD structure described a polyferredoxin (subunit F) docked on electron-transferring subunit G (Figures 1 and 5a). The polyferredoxin harbors six [4Fe–4S] clusters per monomer, totaling an electron network reaching 24 [4Fe–4S] clusters in the tetrameric FMD state,^16^ and was proposed to serve in electron transfer. An accessible peripheral entry point was suspected of performing ferredoxin oxidation and would also allow electron uptakes from artificial sources such as Ti(III) citrate or electrode^1,42^ (Figure 5b). In hydrogenotrophic methanogens, ferredoxins would be mainly reduced by the hydrogenase-containing heterodisulfide reductase, an electron-bifurcating enzyme.^43^ The first structure characterized by the corresponding author in the Shima group illustrated how the H_2_ oxidation by the hydrogenase allowed the concomitant reduction of the heterodisulfide composed of the coenzymes M and B, involved in methane generation (“downhill reaction” *E*°′ = −0.14 V,^44^ recently re-evaluated at −0.28 V^45^), and the reduction of ferredoxin (“uphill” reaction) employed for CO_2_ fixation.^44^ Independent reactions were measured, and the overall electron bifurcation described was shown to be thermodynamically feasible.^43^ Recent studies demonstrated that in some methanogens, FMDs form relatively unstable complexes with the heterodisulfide reductase via the binding of the F subunit.^17^ In this configuration, the uphill reaction of the electron bifurcation reaches a redox potential that is low enough for a thermodynamically favorable formyl–MFR generation. Via an electron bifurcation system coupled to the appropriate downhill reaction (i.e., CoM–S–S–CoB reduction), the initial electron input can be switched to more “classic” donors also used by FDH, such as H_2_, F_420_H_2_, or formate^17,46^ (Figure 5b).

FMDs are not restricted to hydrogenotrophic methanogens and can be employed by other microorganisms to perform the reverse reaction: oxidizing formyl–MFR and releasing CO_2_. While the oxidation reaction coupled to ferredoxin reduction is supposed to ferredoxin reduction is supposed to occur in methylotrophic methanogens and methanotrophs, we recently showed that the FMD system from an ethane-oxidizing archaeon (named *Candidatus* Ethanoperedens thermophilum) couples formyl–MFR oxidation to F_420_ reduction through an electronically connected reductase^2^ (Figure 5). We proposed that the highly favorable coupling is a thermodynamic pull driving anaerobic ethanotrophy,^2^ highlighting the critical role of FMD in this peculiar microbial metabolism.

### 3.2 CO_2_ Electroreduction

The versatility of the electron-transferring module motivated us to investigate FMD possible use for electrode-based CO_2_ electroreduction. Branching enzymes on electrodes is an experimental approach to measuring electron flow (i.e., enzymatic turnover) while providing a stable redox power that is difficult to reach in vitro. The first described enzymatic CO_2_ electroreduction by an FDH published in 2008 proved the feasibility of the approach.^47^ The turnovers obtained under standard temperature and pressure conditions are orders of magnitude higher than those of the artificial catalysts. The reaction is undergone without apparent current loss or generation of side products (e.g., H_2_ or CO). The optimized experimental setup led to efficient enzymatic CO_2_ electroreduction and crucial insights into reaction mechanisms.^36,48−54^

FDHs catalyze formate oxidation at higher rates than CO_2_ reduction,^47^ limiting the accumulated formate concentration at the equilibrium and leading to its oxidation if the electrode potential is unstable (e.g., when coupled to alternative energy sources). In comparison, we tested if FMD specificities would favor formate accumulation by reporting the first FMD-dependent CO_2_ electroreduction in collaboration with the Milton group.^1^ In this work, the enzyme from the thermophilic methanogen *Methermicoccus shengliensis* was natively purified and biochemically characterized. Turnovers similar to those reported for FDHs were measured. Without MFR or the analogue furfurylamine, the electrode-bound FMD reduced CO_2_ with perfect faradaic efficiency, and the formate diffuses out of the enzyme through a yet unclear path. As expected, while the affinity for CO_2_ was similar to that of FDHs, FMD barely catalyzed the formate oxidation, most probably due to the limited diffusion of formate to the active site of the BD subunits.^1^ Furthermore, formate oxidation could not be detected in the presence of an excess of CO_2_, possibly due to a competition effect in the active site. Hence, FMDs are attractive biocatalysts that favor CO_2_ electroreduction without undesired formate oxidation in the case of variation in the electrode potential.

## 4 FMDs as a Source of Inspiration for Biotechnological Applications

The FMD architecture advantages CO_2_ reduction by electrocatalysis, but the enzyme also represents a biological system that synthesizes added-value chemicals directly from CO_2_ by condensation of the produced formate on diverse molecules. Moreover, it presents the advantage of catalyzing relatively unfavorable formylation reactions by the thermodynamic pull from internal formate accumulation. Oxidation of the simpler substrates *N*-furfurylformamide, *N*-methylformamide, and formamide have been reported, yet with catalytic constants orders of magnitude lower than that of the physiological substrate formyl–MFR.^1,55,56^ Hence, it can be expected that FMDs could accept methylamine and ammonium.

**Figure 6:**
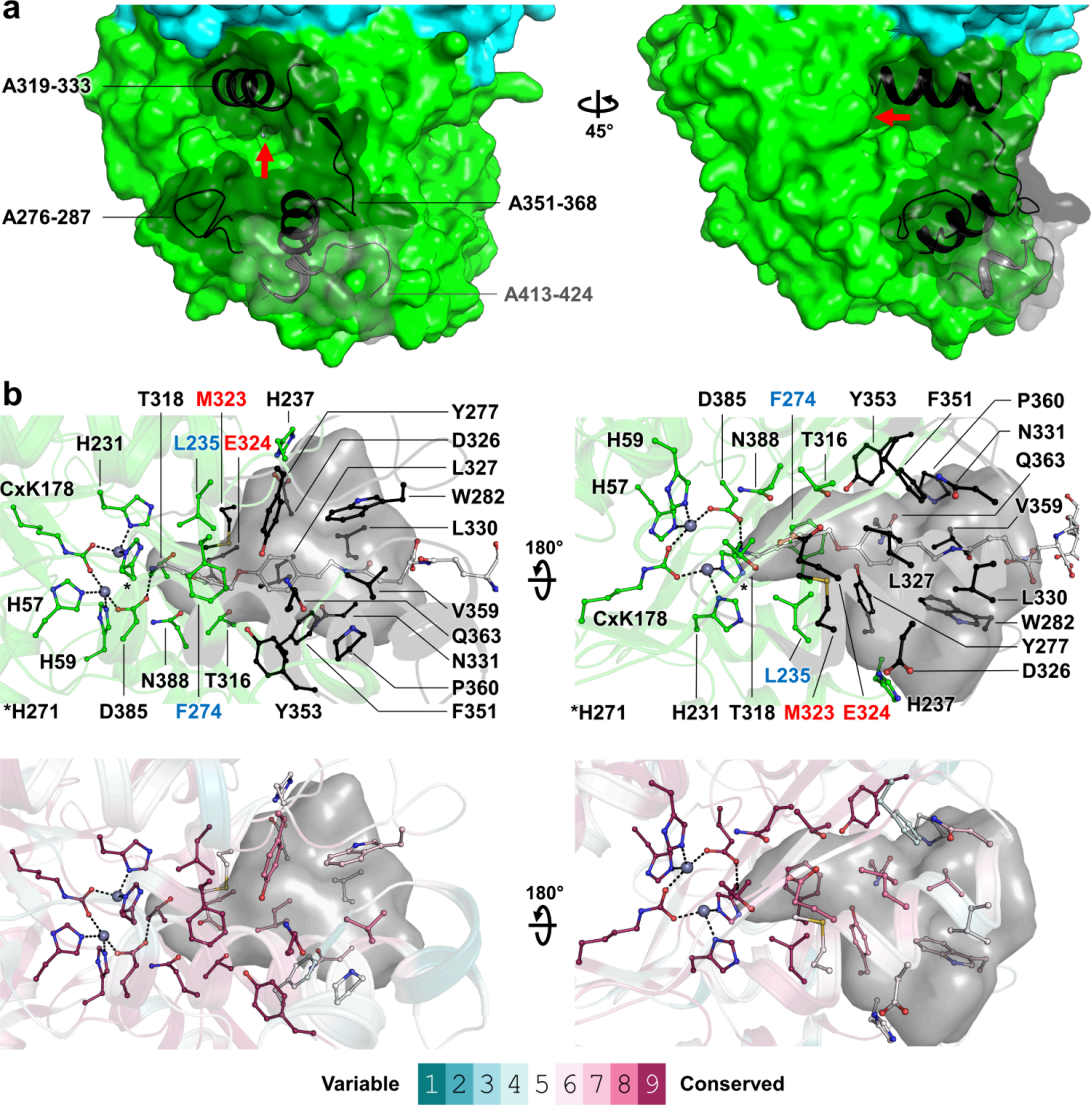
Putative modification sites for the engineering of the A subunit. (a) Structure of the AB subcomplex of the FMD of *M. wolfei* shown as a surface with A and B subunits colored green and cyan, respectively. The loops and helices that could be removed to open the active site cleft are shown as black cartoons and transparent surfaces. A red arrow points toward the access to the [Zn–Zn] binuclear site. (b) Residues conservation and putative substitution sites in the formyl–MFR cavity. The structure is shown as a cartoon, with cofactor, coenzyme, and the residues involved in cofactor and cavity stabilization displayed as balls and sticks and colored as in Figure 1. The formyl–MFR cavity predicted by HOLLOW is a transparent gray surface. The MFR position in the structure of *M. wolfei* (PDB 5T61) is shown in the top panel. The residues are colored as in panel a (top) or by their conservation score (obtained from ConSurf Web server,^65^ bottom) based on the alignment of 96 sequences of A subunits from archaeal FMD sharing 80% sequence identity or less. Proposed targets for substitutions to reproduce amino group stabilization (see Figure 4b) or to enhance the affinity for polar formyl acceptors are labeled in red and blue, respectively (top).

The restricted access to the amidohydrolase active site, highly selective toward MFR due to its hydrophobic constriction, would prevent alternative formyl-accepting amino groups from efficiently diffusing inside and reacting (Figures 6a,b). The residues forming the MFR docking site are conserved among FMDs, suggesting their importance for substrate selectivity (Figure 6b). Enzyme engineering of the amidohydrolase subunit could extend the range of formyl-accepting groups, allowing the synthesis of formamide derivatives. It could be achieved by opening the cleft by removing the surrounding loops and helices (Figure 6a). Hydrophobic residues normally stabilizing the MFR furfuryl group (e.g., Leu235, Phe274, and Met323 in *M. wolfei*) could be replaced with polar residues to enhance the diffusion of small substrates. Specific substitution would redistribute the hydrogen bonding network between the enzyme and the desired substrate, allowing its stabilization in the vicinity of [Zn–Zn] and optimizing activation (Figure 6b). For instance, formamide synthesis would probably be more efficient by mimicking the mechanism observed in urease,^40^ in which a histidine stabilizes the ammonium ion resulting from urea hydrolysis. Reproducing the histidine position in FMD might stimulate formate condensation on the positioned ammonium. Since formate condensation to formamide or *N*-methylformamide is endergonic in standard conditions (+29.4 and +20.9 kJ/mol, respectively^23^), the formate accumulation in the internal cavity must be conserved to drive a favorable overall reaction (see Section 2.1). The enzyme from *M. barkeri*, exhibiting the broadest substrate panel among the studied FMDs,^56^ may be a suitable candidate for engineering even if a more interesting enzyme could be described in the future with the multiplication of studied complexes.

The disadvantages of biological catalysts are their high cost of production and purification, low operation stability, and nonreusability. Despite exhibiting turnover orders of magnitude higher than those of synthetic catalysts, using enzymes for biotechnological applications remains challenging. Because of their complexity, FMDs are usually directly extracted from anaerobic archaea^1,2,16,17,46^ and anaerobically purified through a laborious multistep chromatography process, which is unlikely to be suitable for industrial applications. Overexpression in canonical expression systems (e.g., *Escherichia coli*) would add another challenge because of the maturation machinery necessary for metallocofactors and enzyme biosynthesis. Genetically tractable methanogens such as *Methanosarcina acetivorans* would represent a robust platform to produce and engineer tagged enzymes, simplifying the purification procedure.^57,58^ Large-scale expression by the engineered methanogen could be coupled with methane production to amortize the production costs.

Once obtained, a procedure must be developed to maximize enzyme stability over time. As we have shown, the CO_2_ electroreduction rate of the enzyme bound on electrodes drastically decrease within hours due to its inactivation or release from the electrode.^1^ Additional preparation steps could maximize the enzyme–electrode association, as shown for FDHs.^48^ Enzyme treatment such as encapsulation in an organic framework^59,60^ would probably also enhance the operational stability, albeit the necessity of using a framework conducting electrons to the protein.^61^ As FMDs remain sensitive to oxygen, the operative system would have to be performed with an O_2_ exclusion. Nevertheless, recent works on O_2_-tolerant FDH could inspire the engineering of FMDs to promote their resistance.^39^

FMD could also inspire the rapidly developing field of synthetic biology and artificial enzymes.^62^ Protein scaffolds can be designed to bind extracted biological metallocofactors^63^ or synthetic catalysts.^30^ The development of synthetic catalysts inspired by the pterin cofactor has been attempted for two decades, but reproducing the complicated metal environment is still challenging for chemistry. To date, catalysts still exhibit a relatively poor efficiency (39% of consumed electrons reduce CO_2_ to formate and 14 h^–1^ turnover for a Mo–pterin mimic)^30^ when compared to the highly selective and efficient biological systems (turnover around 850000 h^–1^ for FDH,^48^ with around 100% efficiency). Bio-inspired catalysts from amidohydrolases have been more successful, yet still without matching the efficiency of biological systems.^64^ However, the challenging task of synthetic chemistry remains to reproduce the formate accumulation mechanism to drive the unfavorable reaction of condensation.^15^

**Figure 7:**
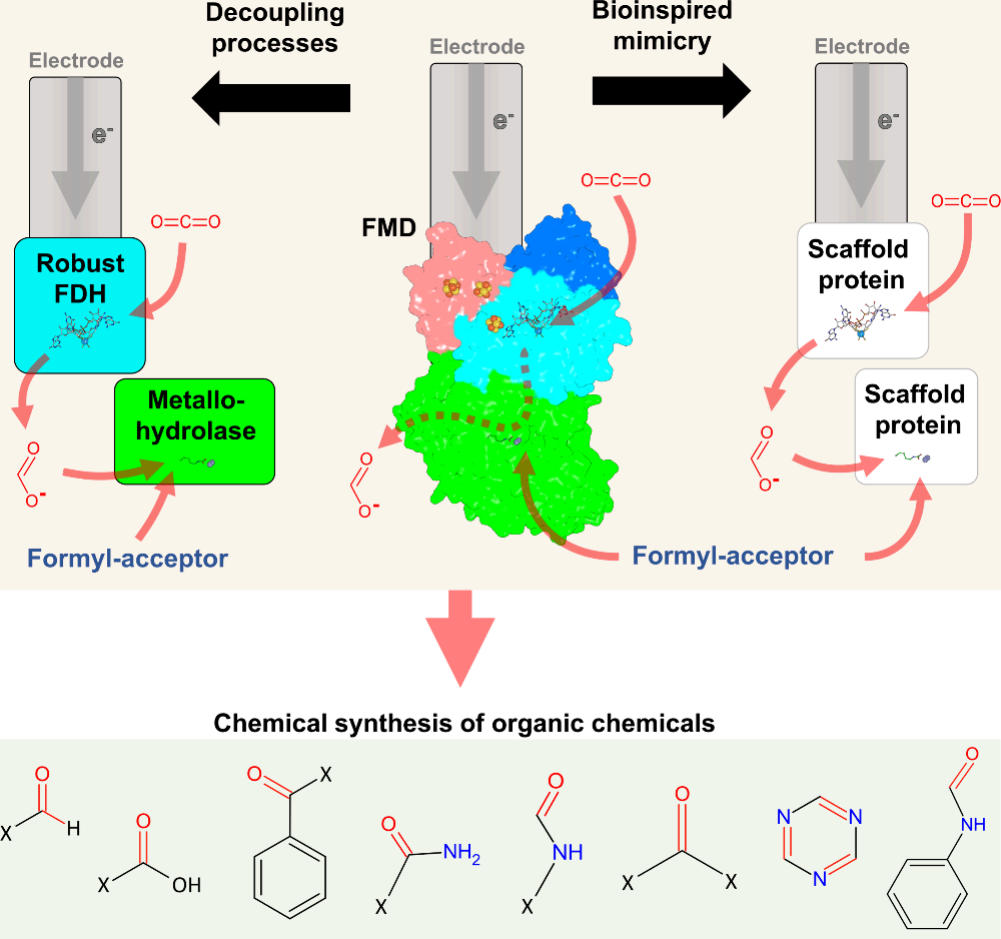
FMD as a biocatalyst and bio-inspiration for C1 conversion. *M. wolfei* FMD structure (PDB 5T5M) is shown as surface and colored as in Figure 1, with cofactors as balls and sticks.

An alternative approach is to use simplified enzymatic systems mimicking FMDs. Robust and O_2_-insensitive FDHs and amidohydrolases (responsible for CO_2_ reduction and formate condensation, respectively) could be produced separately (Figure 7), significantly reducing hurdles and costs in enzyme production. The challenge will then lie in establishing the sufficient proximity of both active sites to mimic the FMD internal cavity and locally reach a high formate concentration necessary for condensation (Figures 1b,c). The strategy might be achieved by encapsulation or protein deposition on a synthetic matrix. Screening a wide panel of FDHs/amidohydrolases and testing their combination would eventually lead to the targeted synthesis of desired final products.

## 5 Concluding Remarks

This Account presents the unique modular organization of FMDs to operate electron transfer to the Mo/W–pterin center, CO_2_ reduction to formate, and the condensation of the latter on the MFR amino group. The corresponding author’s past and present work has elucidated the mechanism of this intricate but elegant biomachinery, which affects the planetary carbon cycle through its role in microbial metabolisms. This remarkable architecture is an advantage for electrochemical processes, as docked (poly)ferredoxins allow a fast electron transfer from the electrode to FMD, resulting in a robust CO_2_ electroreduction turnover with perfect faradaic efficiency.^1^ The formate concentration built up in the internal cavity counterbalances the condensation reaction, granting a favorable thermodynamics of the overall reaction.

Such intrinsic properties place the enzyme at the center of interest as a source of inspiration to transform abundant C1 feedstock CO_2_ into building blocks that can be further processed for organic chemical synthesis (Figure 7). In other words, by turning CO_2_ to formate and formamide, usually extracted from fossil fuel processes, FMD would answer the far-reaching goal of capturing atmospheric CO_2_ while preventing the consumption of fossil fuels. Moreover, its engineering could expand the formyl-acceptor panel to stimulate the production of formamide or derivatives on top of formate. As enzymes’ production cost and operational stability are generally prohibitive for large-scale applications, developing FMD-inspired synthetic catalysts as standalone or concealed in robust scaffold proteins could be the most realistic strategy for developing new CO_2_ conversion applications (Figure 7).
